# Intermodal coupling spectroscopy of mechanical modes in microcantilevers

**DOI:** 10.3762/bjnano.14.13

**Published:** 2023-01-19

**Authors:** Ioan Ignat, Bernhard Schuster, Jonas Hafner, MinHee Kwon, Daniel Platz, Ulrich Schmid

**Affiliations:** 1 Institute of Sensor and Actuator Systems, TU Wien, Gußhaustraße 27–29, 1040 Vienna, Austria

**Keywords:** atomic force microscopy, intermodal coupling, nonlinear mechanics, optomechanics, sideband cooling

## Abstract

Atomic force microscopy (AFM) is highly regarded as a lens peering into the next discoveries of nanotechnology. Fundamental research in atomic interactions, molecular reactions, and biological cell behaviour are key focal points, demanding a continuous increase in resolution and sensitivity. While renowned fields such as optomechanics have marched towards outstanding signal-to-noise ratios, these improvements have yet to find a practical way to AFM. As a solution, we investigate here a mechanism in which individual mechanical eigenmodes of a microcantilever couple to one another, mimicking optomechanical techniques to reduce thermal noise. We have a look at the most commonly used modes in AFM, starting with the first two flexural modes of cantilevers and asses the impact of an amplified coupling between them. In the following, we expand our investigation to the sea of eigenmodes available in the same structure and find a maximum coupling of 9.38 × 10^3^ Hz/nm between two torsional modes. Through such findings we aim to expand the field of multifrequency AFM with innumerable possibilities leading to improved signal-to-noise ratios, all accessible with no additional hardware.

## Introduction

Atomic force microscopy has established itself as one of the most powerful tools in nanotechnology. With meticulous setups amassing techniques such as ultra high vacuum, cryogenic temperatures, and CO-terminated tips, it is able to create a wonderful vista of surfaces, not missing the atoms for the topographical features [1–6]. There is, however, room for improvement in cutting-edge AFM experiments, as the standard quantum limit in sensitivity, represented by a minimum between detection noise and backaction noise, has not been reached [7–8]. Beyond this limit, techniques exist that can even break this quantum barrier by redirecting noise from one quadrature to another [9–11]. Yet, there is even opportunity in revitalising the accessibility of standard AFM, as performing experiments at cryogenic temperatures and under ultra-high vacuum [12–13] requires years of expertise.

For inspiration, we turn to quantum optomechanics and its sister field of quantum electromechanics, as they both report outstanding signal-to-noise ratios [14]. In the former, a reflective mechanical resonator constitutes half of a Fabry–Pérot cavity, converting photons to phonons and vice versa. Thus, the mechanical position can be read through the optical cavity. Upon this basic interaction, many emerging kinds of behaviour were found: sideband cooling down to quantum levels [15–16], parametric amplification [17] before signal detection, state squeezing [18–20], and Bogoliubov modes [21–22] for drastically reducing noise and directional amplifiers [23–24]. The group of proposed applications is even larger and hosts ideas such as quantum circulators [23–24], Ising model simulators [25], and improved gravity wave detection experiments [8]. All these techniques can be migrated to AFM, with the main hurdle being the integration of an optical Fabry–Pérot cavity with an elastic microcantilever. We chose to use purely mechanical coupling, an alternative mirroring our source of inspiration. It relies on non-linear elastic coupling between different vibrational eigenmodes of a mechanical resonator. As the stress field of one mode stiffens the vibrational motion of another, an energy exchange is established between them. This phenomenon is referred to as intermodal coupling [26]. It allows to replace the optical cavity from optomechanics with a mechanical eigenmode.

So far, intermodal coupling was proven in doubly clamped beams, square membranes and circular membranes [18,26–31]. For atomic force microscopy imaging, a slight angle between the sensing mechanical resonator and the sample of interest is required, ensuring that the only contact occurs between the sample surface and the tip of the mechanical resonator. This promotes cantilevers as the chosen geometry for this task, as building a clamped beam or a square membrane at the edge of a chip is considerably more challenging. In the following, we will explore intermodal coupling in a microcantilever as an opportunity to bring intermodal coupling techniques, derived from optomechanics, to AFM. It is easily accessible, with no hardware modifications and only requiring multifrequency excitation applied to the cantilever by either a piezoshaker or a modulated laser, found in many AFM setups.

The field of multifrequency AFM has improved both imaging contrast and the amount of extracted information from AFM experiments by exploiting the nonlinearity of the tip–surface interaction [32–36]. The methods applied excel in both their creativity and engineering prowess. A first example is on-resonance excitation of the first mode of a cantilever with measurements being performed at its harmonics [37]. Another method involved clever designs such as T-shaped cantilevers [38] and inner-paddled cantilevers [39] aiming at reducing the noise impact on force reconstruction. Bimodal AFM is another addition to the field, where two eigenmodes are excited and read simultaneously [40]. Last, intermodulation products, created by two signals close to the fundamental cantilever mode, form a sea of evenly spaced tones to be measured [35,41–42]. All of these rely on the nonlinear tip–surface force to create these multitonal responses, from which the force is reconstructed.

In this paper, we are building towards a hybrid multifrequency approach different from the ones described above. The on-resonance measurement would follow frequency-modulated AFM or bimodal AFM while being assisted by a new off-resonance excitation, which would activate intermodal coupling between two or more eigenmodes. With this geometric nonlinearity, we can circumvent the use of tip–sample forces and apply techniques from optomechanics. Sideband cooling will reduce thermal noise of the fundamental mode. Parametric amplification relies on coherent bimodal drive to amplify the signal of the fundamental mode. Both increase the signal-to-noise ratio of the measurement, creating opportunity for either improved sensitivity or increased speed. Furthermore, sideband cooling has a secondary use in ultrahigh-vacuum AFM as a tool for controlling the *Q*-factor of the fundamental mode.

Intermodal coupling requires a strong drive tone, referred to as a pump, at either the frequency difference between or the sum of two cantilever eigenmodes of interest. Using the difference, also known as a red sideband or anti-Stokes pump, leads to sideband cooling and mode splitting. Applying the sum, referred to as blue sideband pump, will cause either mode squeezing or parametric amplification [22], provided that the amplitude is optimally chosen. We will focus on the red sideband, as sideband cooling is useful for reducing thermal noise in standard AFM and mode splitting is a good way to measure the coupling rates. Here, the phonons from the first mode will have their frequency upconverted to the same as the second mode’s phonons, thus allowing them to interact. This pump effectively amplifies the single phonon–phonon coupling rate of the mode combination and linearly increases the overall coupling strength 
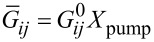
, where *X*_pump_ is the pump amplitude, thus giving us the following Hamiltonian for one eigenmode *i* coupled to another eigenmode *j*


[1]

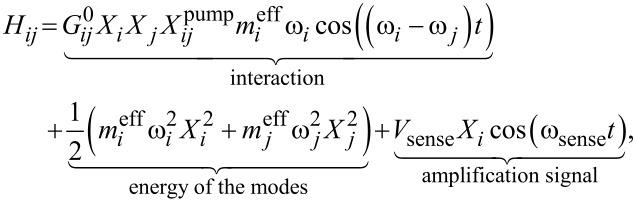



where ω*_i_* and ω*_j_* are the frequencies of the *i*-th mode, henceforth known as the sense mode, and *j*-th mode, taking the role of the cavity mode in cavity optomechanics, respectively. *X_i_* and *X_j_* are their respective amplitudes, 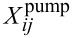 is the amplitude of the pump in meters, 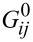 is the directional single phonon–phonon parametric coupling rate in Hz/meters. The last term describes a small signal *V*_sense_, proportional to the voltage applied to the piezoshaker, with the frequency swept close to ω*_i_*, used to amplify the spectral response of the sense mode above the thermal excitation level.

The above Hamiltonian is a modified version of the one used in [27]. In contrast to this previous work, we do not exclude the possibility of asymmetrical coupling. This refers to an energy transfer either easier or harder from the first mode to the second compared to a transfer from second to first. Two directional coupling terms were introduced to account for this possibility, later to be investigated in detail. Equation 1 only shows the energy of two modes and their interaction, amplified by the red sideband pump, which is set at the frequency difference of the two modes in question. A main advantage of working with continuous mechanical systems, such as microcantilevers, is the plethora of eigenmodes available [43]. For every combination of two eigenmodes, a pump frequency can be applied to activate that intermodal coupling. Thus, the Hamiltonian can be expanded to include more eigenmode combinations including their individual energies as well as the interaction terms (the latter is only relevant if a pump is applied). We will focus only on a finite number of eigenmodes due to our equipment limitations. The full Hamiltonian is given by


[2]

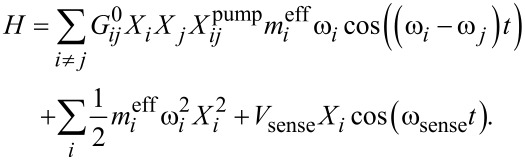



If this coupling is a direct analogue to optomechanics, the coupling matrix should be symmetric, that is, 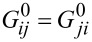. Expanding the experiment to multiple eigenmodes will elucidate if this symmetry is respected or not in these purely mechanical interactions and provide a spectroscopy map of intermodal coupling.

The coupling presented so far, using a red sideband signal, has two ways for manifesting itself, namely sideband cooling, where the mode of interest has its quality factor reduced alongside its effective temperature, and mode splitting, where two hybridized eigenmodes replace the original. The latter is useful in estimating the coupling strength, but the former is more applicable to AFM. It can not only control the quality factor of cantilevers, but it can also reduce the thermal noise of the measurement. These two kinds of behaviour have a regime associated to each, both directly related to the overall coupling strength 
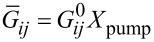
. The *i*-th mode, as the sense mode, is in the weak regime if 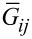 is smaller than Γ*_j_*/2, the linewidth of the cavity mode. In this case its susceptibility (spectral response) can be written as


[3]

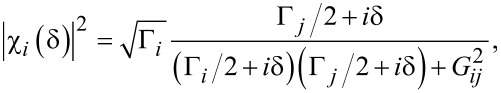



where δ is the frequency offset from the eigenfrequency ω_1_, and Γ_1_ and Γ_2_ are the linewidths of the modes. The equation can be further simplified to a Lorentzian with an increasing effective linewidth as per equation 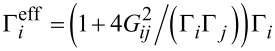, enabling us to extract the coupling strength. If *G_ij_* *>* Γ*_j_*/2, the sense mode is in the strong regime. Here the susceptibility equation is


[4]

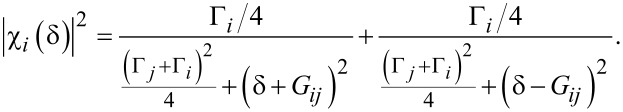



In this case, the distance between peaks can be approximated as 
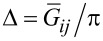
.

The effective temperature of the mode is calculated by normalizing the integral of the measured amplitude squared to the case when the pump is off when the system is at room temperature as follows:


[5]

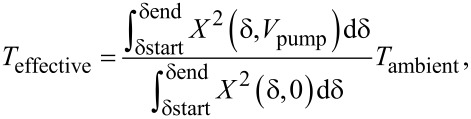



where *X* is the spectral response amplitude with respect to the frequency offset from eigenfrequency δ and pump amplitude *V*_pump_, *T*_ambient_ is the temperature of the room where the experiment was performed, and δ_start_ and δ_end_ are the start and end frequencies, respectively, of the lock-in measurement.

**Figure 1:**
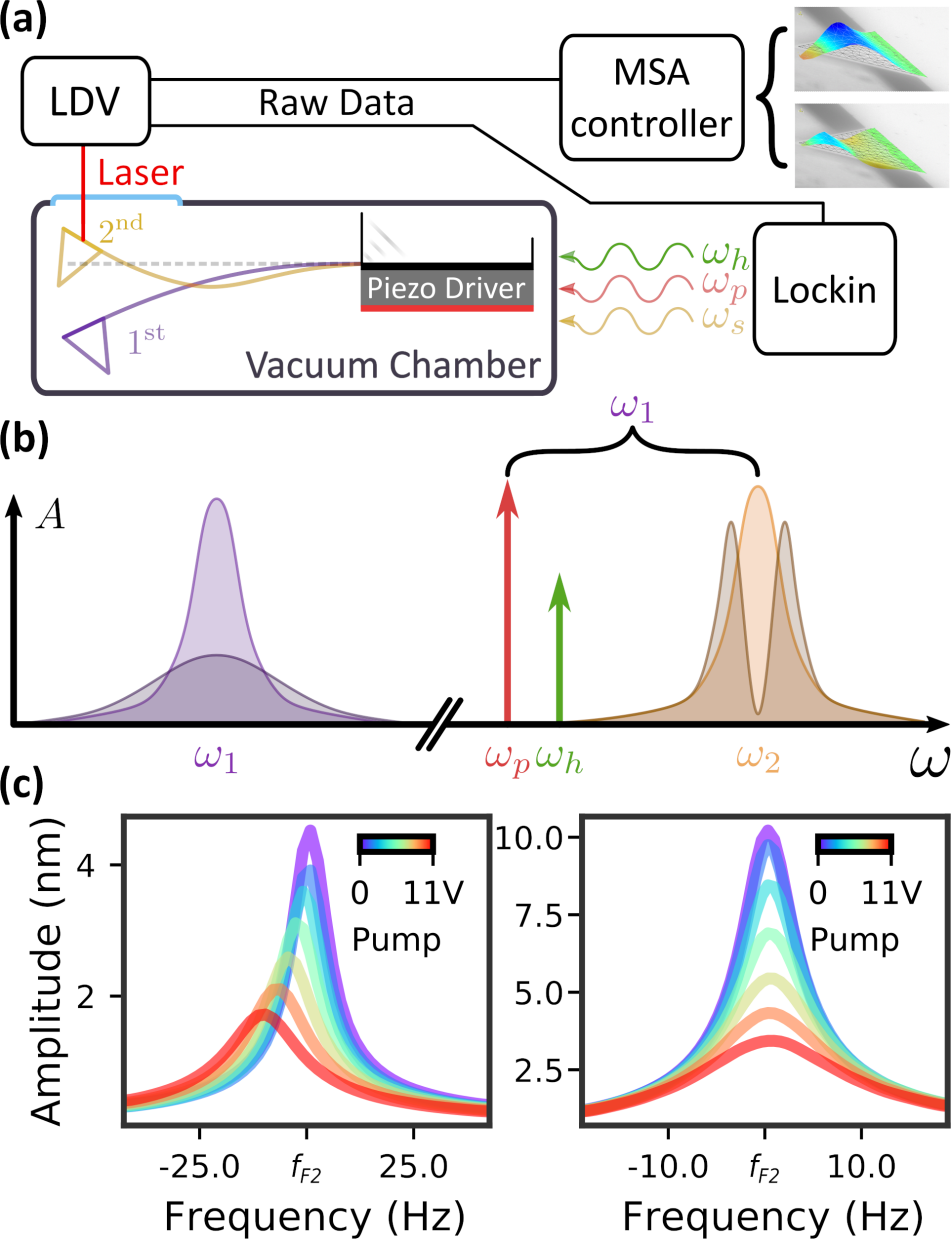
(a) Schematic drawing of the experimental setup. The cantilever is glued to the macrosized piezo driver. The LDV can either send data to the MSA to determine the eigenmode shapes or to the lock-in amplifier for higher bandwidth measurements. The latter also synthesizes the signal applied to the piezo driver. (b) Schematic of the signals used. Three signals are in effect at all times: the red sideband pump ω_p_, an off-set red sideband pump ω_h_ ensuring even heating across the sample and a small one, compared to the previous, sweeping over the sense mode. (c) Comparison between a two-signal measurement (left) and a three-signal measurement (right) ensuring thermal stabilisation. The second flexural mode is coupled with the fifth flexural mode. The sum of heating signal and pump is constant. The stabilisation signal was 3 kHz higher than the red sideband pump, which was set at 3176.9 kHz. Due to their large frequency distance from the observed mode compared to its linewidth, ω_p_ and ω_h_ were not included in the graph.

An AFM microcantilever (Bruker RFESP-75) is glued to a piezoshaker and placed in a vacuum chamber (between 1.2 × 10^−6^ and 5 × 10^−7^ mbar) under a laser Doppler vibrometer (LDV) (Polytech MSA 500) to measure the cantilever’s resonance frequencies and mode shapes (Figure 1). A lock-in amplifier (Intermodulation Products MLA-3 [36]) is used to control the piezoshaker and measure multiple frequencies from the vibrometer. For each possible mode combination, we activated the anti-Stokes pump and used a smaller sweeping signal to amplify the sense mode.

## Results and Discussion

**Figure 2:**
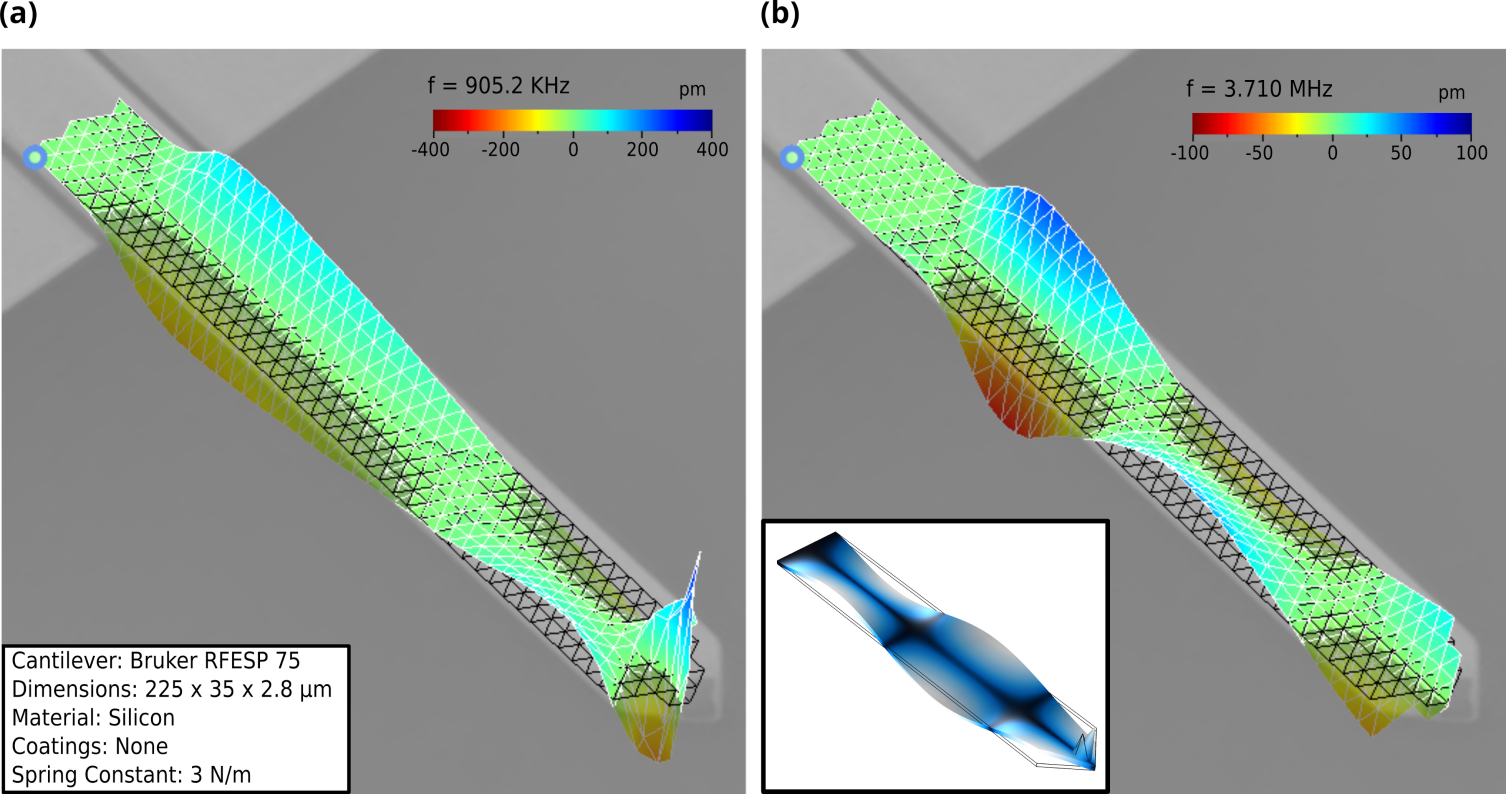
MSA measurements showing the difference in modeshapes between the third order torsional modes investigated in the main text. (a) is T3’ with a node much closer to the added mass of the tip. (b) is T3, with nodes closer to their expected positions. Inset: FEM simulation of T3 eigenmode.

Compared to a plain microcantilever, one with an AFM tip has certain peculiarities to it. Table 1 shows the eigenmodes and their frequencies of the modes of interest in the cantilever used, measured using a LDV. Alongside it, in the last column, we provide FEM simulation estimations for the frequencies. The appearance of multiple torsional modes of the same order was observed experimentally on multiple cantilevers, but could not be replicated with a simple FEM model. Figure 2 shows a comparison between the two third-order torsional modes present in the cantilever (T3 and T3’). The anomalous one, T3’, unseen in the FEM simulations, has the nodal lines much closer to the added mass. The other orders were observed below the frequency of T3’, but they were much harder to excite with the piezoshaker used for the experiment and, therefore, excluded from the analysis. The existence of these modes can be explained through a combination of the extra mass of the AFM tip on the cantilever and material differences in the silicon caused by fabrication processes.

**Table 1:** Table showing the eigenmodes and their frequencies accompanied by the *Q* factors of the modes used in the study. The cantilever investigated is a Bruker AFM RFESP-75. Measurements were performed at pressures between 1.2 × 10^−6^ and 5 × 10^−7^ mbar, a range where both frequencies and quality factors were stable.

Eigenmode	Frequency (kHz)	*Q* factor	FEM frequency estimation (kHz)

first flexural (F1)	62.026	106149	62.176
second flexural (F2)	390.320	57227	388.35
first torsional (T1)	701.158	113437	704.17
anomalous third torsional (T3’)	905.237	3324	–
third flexural (F3)	1096.585	3974	1085.5
second torsional (T2)	2146.963	32469	2150.2
fourth flexural (F4)	2154.353	6259	2122.9
fifth flexural (F5)	3567.223	3842	3497.8
third torsional (T3)	3710.387	46290	3703.6

After determining the modes available for measurement in the cantilever, we can focus on interaction between any two modes. Once a combination of modes is chosen, we focus on each mode separately as the sense mode. We measure the resonance frequencies just before performing the experiment, thus excluding shifts caused by vacuum changes or temperatures fluctuations. Such a technique can be performed using a phase-lock loop when employed in AFM sensing, where tip–surface forces would cause frequency shifts. We stabilise for any heating effect caused by the high-voltage pump applied to the piezoshaker by adding a temperature stabilisation tone with an offset of around 3 kHz, or more if the linewidth of the sense mode becomes comparable. This second pump is set up such that it does not amplify the intermodal coupling, as the chosen offset is larger than all linewidths observed during the investigation. Thus, any products of the pump and another eigenfrequency would not coincide with another eigenmode. This temperature stabilisation tone does have a very similar heating effect as the red sideband pump. Keeping the sum of the voltages applied to the piezoshaker constant, will ensure that the heating power introduced in the system does not change when increasing the pump amplitude. Note that the amount of heating depends on both the piezoshaker used and the frequency of the signal applied. Figure 1c shows an example on the effects of such a stabilisation approach, where the eigenfrequency does not shift to lower values due to thermal length extension of the cantilever. Next, we send a small frequency sweeping signal to measure the susceptibility of the sense mode.

**Figure 3:**
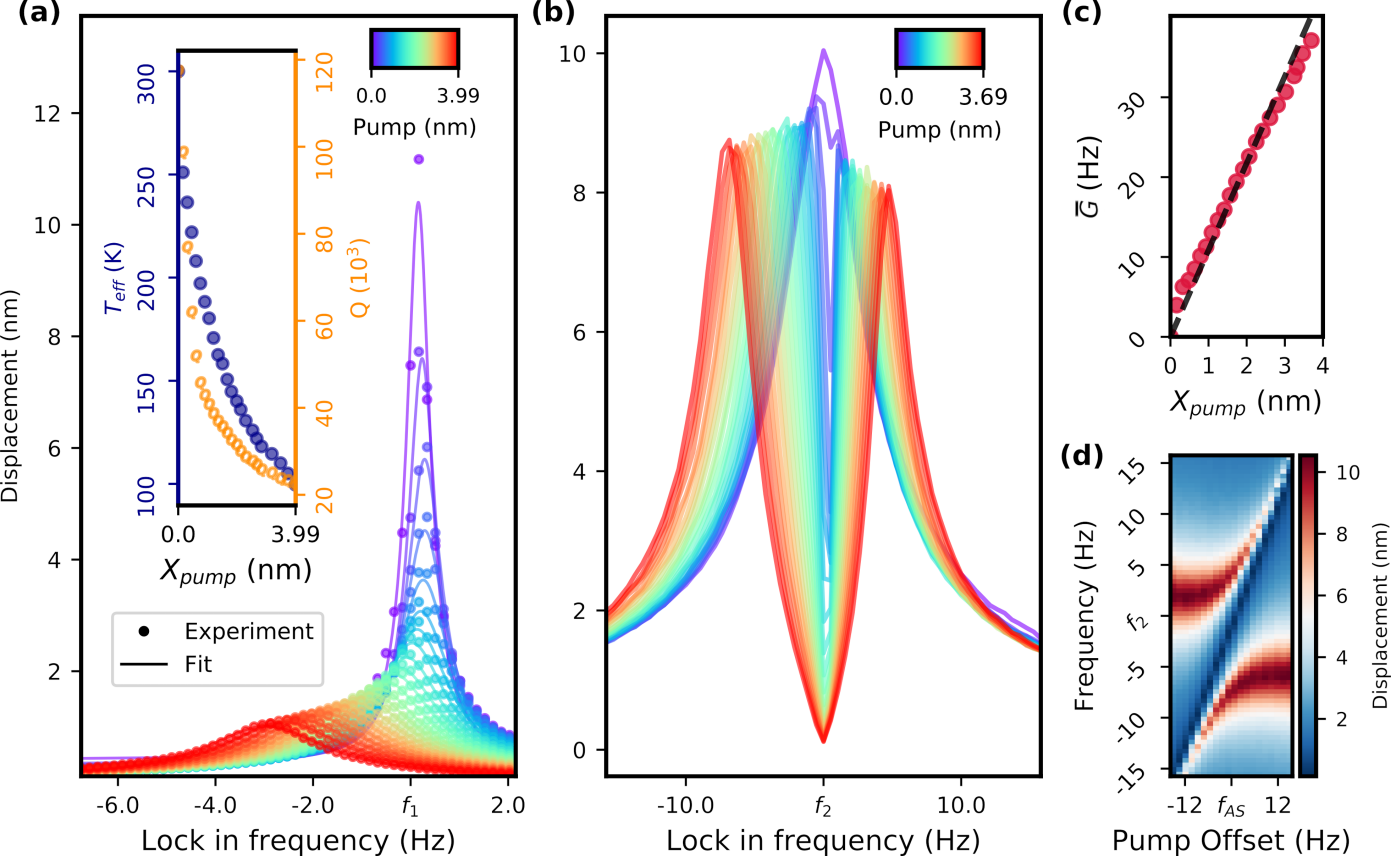
(a) Measurements of the first mode coupled with the second. Increasing the pump amplitude presents both a shift in the frequency and a reduction in effective temperature. Inset: Effective temperature and *Q* factor as functions of the pump amplitude. (b) Data of the second mode under different pump settings. Mode shapes under increasing amplitude of the pump. (c) Estimation of the coupling strength from data in (b). Slight deviations from the linear fit are caused by the approximation used. (d) Colormap of second mode for different frequency offsets of the pump at fixed amplitude. *f*_AS_ refers to the anti-Stokes pump frequency.

First, we investigate the first possible mode combination on our cantilever, that is, the first and second flexural modes. In Figure 3a, we sweep a small signal across the first mode. Each line was measured for a single value of the pump amplitude. As the amplitude of the pump increases, the linewidth does as well while the amplitude decreases as per Equation 3. We calculate the effective temperature using Equation 5 and we achieve a reduction down to just below 100 K. The results of this evaluation are seen in the inset of Figure 3a. This data set also exhibits a significant frequency shift, as it was measured without the thermal stabilisation technique described above.

Keeping the pump constant while sweeping the signal tone over the second mode, we have an example of the strong coupling regime, seen in Figure 3b. As soon as the pump is turned on, there are two distinguishable hybridized eigenmodes in lieu of the original. Increasing it further ensures that the two peaks are resolved, as the local minimum in the middle decreases and the two maxima drift further apart. The coupling strength is estimated using the frequency difference between the two peaks, as in the approximation in Equation 4, and presented in Figure 3c. With the current setup we achieved a coupling rate of 37.1 Hz. Deviations from the linear fit line starting from the origin are a direct consequence of the approximation. It forgoes the interference between the hybridized modes around the original eigenfrequency, which pushes their peaks further apart the closer they are. Therefore, coupling values at lower pump amplitudes are overestimated. Figure 3d shows an amplitude colormap of the same mode for different frequency detunings of the anti-Stokes pump. The higher the detuning, the greater the difference in amplitude between the two peaks. As expected from an avoided mode crossing, the distance between the two hybridized eigenmodes is minimal when the pump frequency equals the frequency difference between the resonance frequencies of the modes. For the rest of the data we readjusted this frequency by repeating lock-in measurements of the eigenmodes whenever necessary to avoid any issues caused by daily thermal drift.

**Figure 4:**
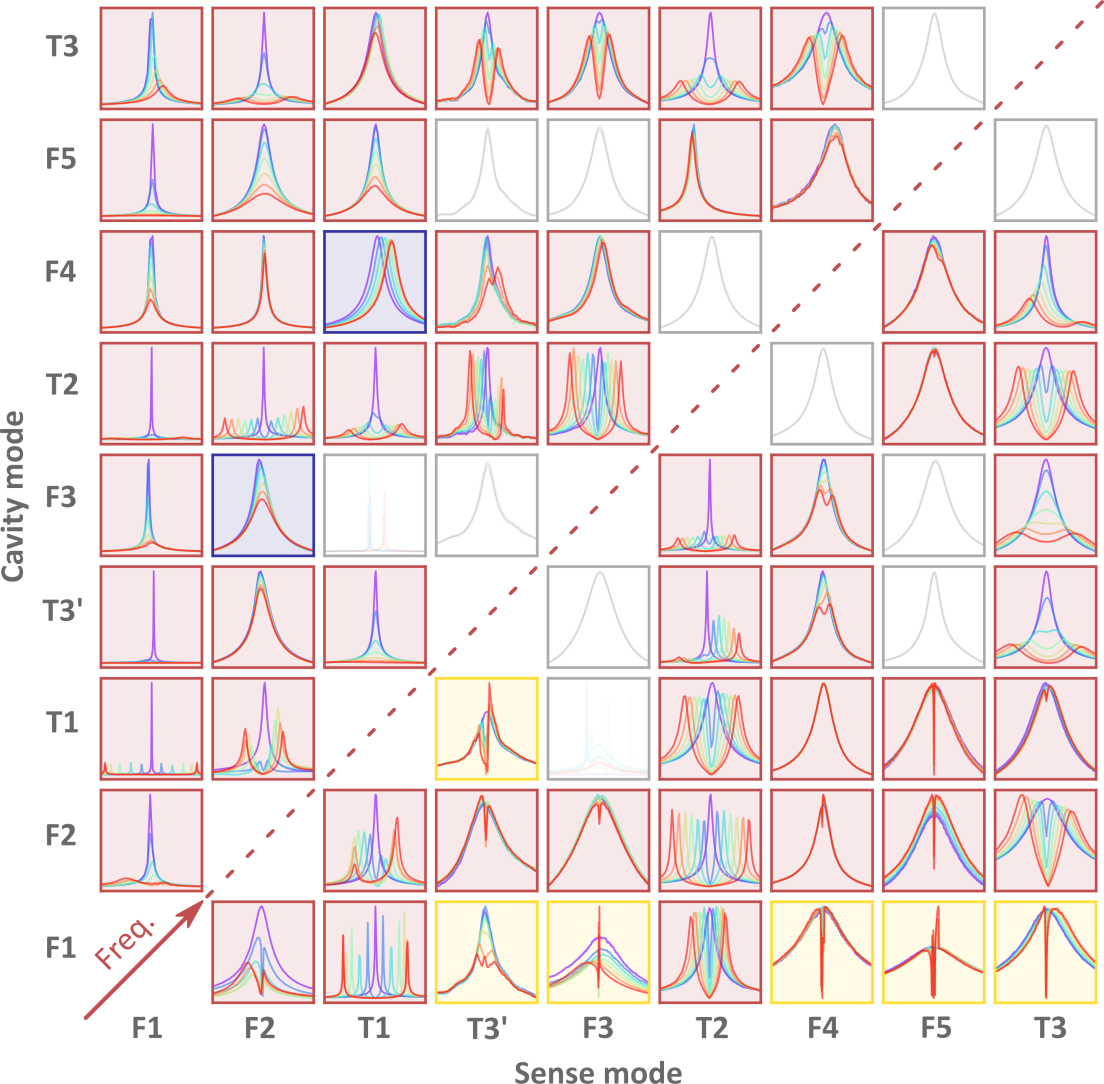
Map of the observed modes under anti-Stokes pumps. On the columns we have the sense mode, while the rows designate the mode it is coupled to, from bottom left. The greyed out graphs are setups where no discernible coupling is present. The red ones follow the expectation of the optomechanical Hamiltonian. The yellow ones exhibit nonlinear behaviour not described by the aforementioned Hamiltonian. Blue have a significant frequency shift, on the same order of magnitude as the linewidth of the sense mode, unexplained by cantilever expanding under heating.

**Figure 5:**
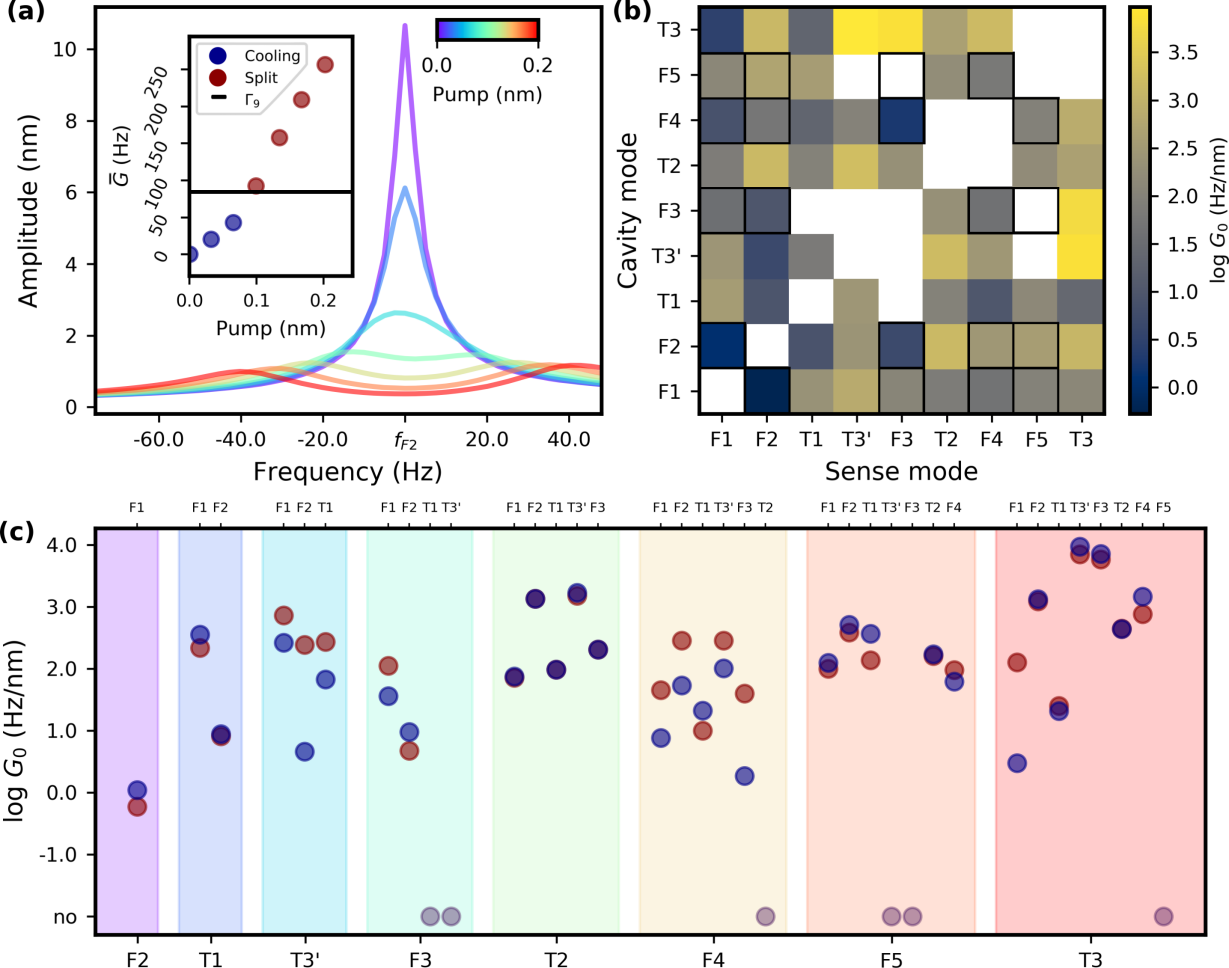
(a) Graph for mode combination F2–T3, which has a regime transition. Inset: Coupling rates determined from linewidth changes or eigenmode separation against half the linewidth of cavity mode T3. (b) Matrix showing the coupling rates of all mode combinations. Contoured squares represent combinations between flexural modes only. (c) Same data as in (b) presented in a one-dimensional perspective. Blue points are calculated from data sets with the sense mode lower in frequency than the cavity mode, while red are the opposite. Greyed out points have no discernible coupling.

The applications we envision for AFM benefit from stronger coupling rates. Therefore, we extend these measurements to the first nine modes of the cantilever under test. Figure 4 shows both the lower and the higher frequency mode response of each possible combination. Coupling rates are calculated from the distance between the two hybridized modes, the increasing linewidth, or both if a regime change from weak to strong can be seen, which is the case for F2–T3 (i.e., sense mode F2 with cavity mode T3). This specific case is explored further in Figure 5a with an inset detailing the coupling rate values taken from the two regimes. The split measurements are overvalued due to the approximation as described previously. The inset has a horizontal line at half the linewidth of the cavity mode. The regime changes at this point as detailed before. Figure 5b presents the coupling matrix and a colormap containing the directional coupling strength between two modes normalised to pump amplitude in nanometers. The highest measured coupling rate between flexural modes is 5.15 × 10^2^ Hz/nm. Overall, T3 and T3’ showed an even higher *G*^0^ at 9.38 × 10^3^ Hz/nm. For comparison with literature values, we need to see the dependence of the coupling strength on the pump voltage used. For the same mode combination presented above, the coupling strength achieved is 5.49 × 10^2^ Hz/V, greater by a factor of 3.4 compared to other findings [28]. Exploring the coupling map further, one can observe that, for flexural modes, the higher the order, the higher the coupling strength per nanometer of pump amplitude. Mode combinations that include torsional modes also exhibit the same effect.

The map is mostly filled, although there are several exceptions with no indication of coupling. There are multiple explanations for the empty spaces, and all can have an impact on the lack of coupling. First, a piezoelectric actuator can have a minimum in its response function at the pump frequency. Second, the intermodal coupling effect can be at a minimum in these combinations. Last, any visible effect might be obscured by daily thermal fluctuations and the finite time for measurements that they impose.

Coming back to the question of coupling symmetry between two modes, Figure 5c shows the same data as Figure 5b but in a folded perspective. Blue points represent data from lower-frequency sense modes in the combination, while red points show the opposite. Out of 30 combinations exhibiting intermodal coupling, 19 show symmetry. Furthermore, amongst the eleven that do not present symmetric results, nine have a higher value for the coupling rate extracted from splitting data. Eight of them are far away from the approximation of two separated Lorentzians for the peaks. Improvements can be made by fitting Equation 4, which lowers the estimated values for *G_ij_*. This requires better temperature control to ensure that no shifts occur during the pump application and the aforementioned equation applies. The piezoshaker has a different heating response with respect to the signal frequency. Equation 4 requires an anti-Stokes pump with a perfectly tuned frequency. Bringing everything in frame, there are more points that have symmetry than not. This does not exclude the possibility that some mode combinations do exhibit asymmetric coupling mechanisms. Beyond the assumed interaction Hamiltonian, terms of different orders might apply.

During our investigation, nonlinear interactions were observed and presented in Figure 5 as the yellow or and blue graphs. Peculiar deviations from the strong regime theory can be seen in the combinations T3’–F1, T3’–T1, F3–F1, F5–F1, and, to a lesser extend, in F4–F1. The effect becomes more pronounced at higher pump amplitudes, where new peaks begin to appear in the vicinity of the local minimum. This might be caused by an excitation of the cavity mode either due to proximity to the pump signal or electrical sideband of the sense and the pump signals. Another possibility is an eigenmode not within the combination being excited by the red sideband pump, leading to a pump amplitude comparable to the sensing amplitude, or to one harmonic of a different eigenmode being excited. This might be the case for F3–T1 with the pump close to F2 (1.013) or for F5–F1, where the pump is close to the fifth harmonic (4.9992) of T3’. The other combinations exhibiting this behaviour did not have the pump aligned with any known mode or harmonic. Both lead to an unstable regime for the amplitude of the cavity mode. Having another eigenmode as the pump was slightly explored before [18], yet its linewidth was not taken into consideration.

Another nonlinear effect can be observed in T3–F1. Here, the local minimum decreases with the pump as expected, yet the two hybridized peaks are asymmetric in their lineshape. The one on the left exhibits a shear drop in amplitude towards the dip, while the right one misses such feature.

Last, T1–F4 has a frequency shift. This is not uncommon in the measured data as F1–T3, F1–F3, and F3–F4 show it as well. Heating effects would cause a quadratic shift with respect to the pump voltage, dominated by the thermal length extension of the cantilever, either up or down due to the extra signal used for compensation. In contrast, the frequency shift of T1–F4 is linear. A cause of this can be a different coupling term of higher order involving the mode energies directly. The same effect might be found in F2–F3 alongside a significant quadratic heating effect, causing a maximum in the frequency shift.

Throughout these measurements, the sensing voltage was carefully tuned as to not bring any of the modes in the Duffing regime. As the amplitude of the mode is increased, Duffing nonlinearity is recognized by an asymmetric modeshape and a frequency shift, followed by an eventual bistable oscillation. This is characterized by a nonlinear term in the oscillator equation proportional to the cube amplitude.

## Conclusion

We investigated the purely mechanical coupling capabilities of a typical AFM cantilever. For this purpose, we used a pump set at the frequency difference between two mechanical modes of interest. Repeating the procedure for all possible combinations of the observable eigenmodes creates a modal coupling map of the microresonator. Each combination is calibrated to its amplitudes in nanometers to reveal preferable combinations as well as incompatible ones. Such a data set alongside knowledge of the eigenmodes themselves can help us reveal the nature of intermodal coupling. Most of the intermodal coupling data points support a symmetric coupling Hamiltonian similar to the one used in optomechanical systems. This will inevitable lead to engineered microresonators taking full advantage of this phenomenon.

Mapping these couplings allows one to activate multiple couplings at the same time. Innumerable applications include those studied in optomechanics and electromechanics, as well as theoretical implementations yet to be seen in practice, all powered by phonon–phonon interactions. Not only bringing improvements to common AFM tools, but providing opportunities for higher sensitivities in cutting-edge AFM as well.

One important aspect of the presented work is the quantitative coupling spectroscopy and the amount of strong evidence towards symmetry in coupling between cantilever modes, further enforcing the link between this intermodal coupling and its inspiration, optomechanical coupling. Such insight contours a vital step for the development of novel multifrequency methods by allowing one to distinguish coupling effects that are mediated by nonlinear elasticity or tip–surface interactions. Inclusion of intermodal coupling would pave the way for a new era of multifrequency AFM methods designed for controlling the sense mode to reach their ultimate goal of greater signal-to-noise ratios.

These possibilities only multiply if the mechanical–mechanical interactions were only one aspect of a device. In a MEMS or NEMS device, such interactions would be useful to bridge electrical modes together, opening up the possibility of creating transducers mediated by a moving capacitor. Such thoughts open the doors to sensors with qualities overshadowing their predecessors.
